# Repeated detection of West Nile virus in *Aedes caspius* during an unusually early transmission season in humans in northern Italy, 2026

**DOI:** 10.2807/1560-7917.ES.2026.31.42.2600862

**Published:** 2026-10-22

**Authors:** Mario Chiari, Francesco Defilippo, Davide Lelli, Luigi Vezzosi, Danilo Cereda, Gabriele Del Castillo, Francesco Maraschi, Angela Ancona, Giuliano Rizzardini, Anna Marelli, Ambra Nucci, Moira Bazzucchi, Maria Gabriella Perrotta, Marino Faccini, Marinella Valenti, Simone Villa, Davide Mileto, Alessandra Lombardi, Alessandro Mancon, Valeria Micheli, Andrea Gori, Stefania Piconi, Greta Romano, Guglielmo Ferrari, Antonio Piralla, Francesca Rovida, Ana Moreno, Fausto Baldanti

**Affiliations:** 1 General Directorate of Welfare, Regione Lombardia, Milan, Italy; 2 Istituto Zooprofilattico Sperimentale della Lombardia e dell’Emilia-Romagna (IZSLER) “Bruno Ubertini”, Brescia, Italy; 3 Lombardy Regional Center for Infectious Diseases (CeReMI), Milan, Italy; 4 National PhD Programme in One Health approaches to infectious diseases and life science research, Department of Public Health, Experimental and Forensic Medicine, University of Pavia, Pavia, Italy; 5 Direzione generale della sanità animale e dei farmaci veterinari, Ministero della Salute, Rome, Italy; 6 Department of Hygiene and Public Health Prevention, ATS Milano Città Metropolitana, Milan, Italy; 7 Department of Veterinary Medicine and Food Safety of Animal Origin, ATS Milano Città Metropolitana, Milan, Italy; 8 Clinical Microbiology, Virology and Biomergencies, ASST Fatebenefratelli-Sacco, L. Sacco University Hospital, Milan, Italy; 9 Infectious Disease Unit, ASST Fatebenefratelli-Sacco, L. Sacco University Hospital, Milan, Italy; 10 Infectious Disease Unit ASST Lecco, Lecco, Italy; 11 Microbiology and Virology Department, Fondazione IRCCS Policlinico San Matteo, Pavia, Italy; 12 Department of Clinical, Surgical, Diagnostic and Pediatric Sciences, University of Pavia, Pavia, Italy; * These authors contributed equally to this work and share first authorship

**Keywords:** West Nile virus lineage 2, Aedes caspius, Culex pipiens, mosquito surveillance, Lombardy, Italy

## Abstract

In Lombardy, northern Italy, 2026, the first neuroinvasive West Nile virus (WNV) human case was reported on 7 July. Concurrently, high abundances of *Aedes caspius* and low numbers of *Culex pipiens* occurred at several surveillance sites. Testing seven archived *Ae. caspius* pools collected in July revealed WNV lineage 2 in six pools from three independent sites. A virus sequence from *Ae. caspius* shared > 99.94% nt identity with contemporaneous human-derived sequences. Further investigating possible *Ae. caspius* vector competence, particularly for WNV lineage 2, is warranted.

In 2026, West Nile virus (WNV) activity in Lombardy started unusually early with some initial human cases reported in the west. The first human neuroinvasive case was reported in Mortara (Pavia Province) on 7 July, followed by a second case in Abbiategrasso (Milan Province) on 10 July. Concurrently, an unusual shift in mosquito community species composition was observed at several surveillance sites in the western part of the region, with a marked increase in *Aedes caspius* and a decrease of *Culex pipiens* abundance. After detecting WNV-positive mosquito pools, we investigated the mosquito species involved in viral circulation and assessed WNV occurrence in *Ae. caspius*, a species in which WNV RNA has prior been detected, but for which current evidence does not clearly establish a role as a competent vector or an involvement in human transmission [1-3].

## Description of the 2026 West Nile virus season in Lombardy

During July 2026, human WNV infections were reported in several provinces in Lombardy. Between 7 and 20 July, individuals with West Nile neuroinvasive disease (WNND) were identified in Pavia, Milan and Lodi provinces, while WNV-positive blood donors were detected in multiple provinces (Figure 1A-B).

**Figure 1:**
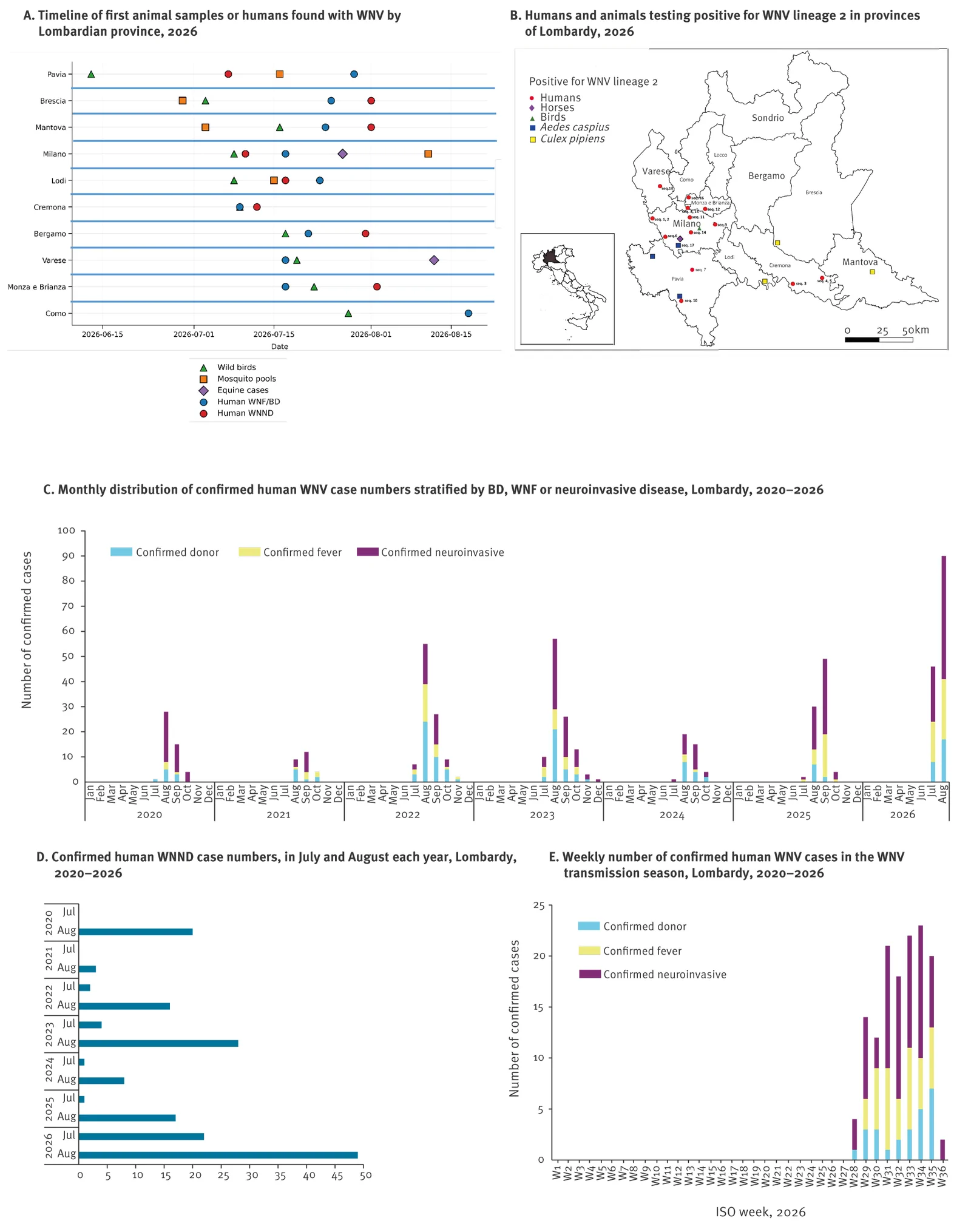
Spatiotemporal distribution of West Nile virus detections and, in context of 2020–2026 epidemiological trends, human infections in Lombardy Region, Italy, 2026

BD: blood donors; ISO: International Organization for Standardization WNF: West Nile fever; WNND: West Nile neuroinvasive disease; WNV: West Nile virus.

A. Timeline in respective provinces of the Lombardy Region, Italy, 2026, of first sampling or suspicion leading to the detections of WNV by surveillance component (i.e. wild bird, mosquito and horse). Times of first human WNV infections are also shown. Dates for wild birds, mosquito pools, and equine cases correspond to sampling or suspicion dates, whereas dates for human infections correspond to notification dates. Human infections are classified either as WNND or as non-neuroinvasive infections, including WNF and infections detected in BDs.

B. Geographical distribution of WNV detections in mosquitoes, wild birds, horses, and humans across the provinces in Lombardy found affected by the 2026 WNV transmission season. For detections associated with viral sequences included in the phylogenetic analysis, the corresponding sequence identifier (Seq + number) is reported on the map to facilitate cross-referencing both with the Supplementary Table 1 describing the sequenced samples’ characteristics and with the phylogenetic tree (Figure 2). On the map, each red dot represents one human WNV case, while each blue square represents a mosquito surveillance site where one or more mosquito pools tested positive for WNV lineage 2.

C. Monthly distribution of confirmed human WNV cases by classification (BD, non-neuroinvasive disease, and WNND) in Lombardy Region, Italy, from 2020 to 2026.

D. Monthly distribution of confirmed WNND cases from 2020 to 2026.

E. Weekly distribution of confirmed human WNV cases by classification during the 2026 transmission season (ISO weeks 20–36; data up to 31 August 2026).

By 19 August, a substantial increase in the number of reported human WNV cases had occurred. In Milan Province, the province with the highest number of human cases, this had reached 41, including 26 WNND (63%), compared with 30 cases and 16 WNND (53%) during the entire 2025 season (Figure 1C-E). For Lombardy, the temporal distribution of confirmed human WNV cases during 2026, in comparison with previous seasons, is presented in Figure 1 C-E.

Concurrent veterinary surveillance, including avian and entomological monitoring, confirmed widespread circulation of WNV in Europe [1,4,5]. In Lombardy, the geographical distribution of WNV-positive birds, equines, mosquitoes and humans is shown in Figure 1B. In the Milan province, the first WNV-positive wild bird was detected on 8 July and by 19 August a total of 28 infected wild birds had been identified, including *Turdus merula*, *Athene noctua*, *Pica pica* and *Corvus cornix*. After 19 August, a clinical equine case was subsequently confirmed in Vermezzo con Zelo. 

Across Lombardy, entomological surveillance detected WNV-positive pools of *Cx. pipiens*, the primary targeted mosquito species monitored for the virus. These observations occurred within the Lombardy regional mosquito surveillance network established under the Italian National Arbovirus Plan [6]. Surveillance sites included Villachiara in Bescia Province, Rosate in Milan Province and Parona and Voghera in Pavia Province, all in areas with previous evidence of WNV circulation. While the first positive mosquito pool identified in the region was in Brescia Province on 29 June, in Milan Province the initial positive pool was detected on 11 August.

## Entomological investigation

Although surveillance traps are designed to collect *Cx. pipiens*, of which pools are routinely tested throughout the season, they also capture other mosquito species, particularly *Ae. caspius*. Specimens of this non-target species are usually stored and tested only at the end of the season.

In Milan Province during June–July 2026, however, a marked shift in mosquito species composition was observed at surveillance sites. Overall, the number of *Ae. caspius* specimens found in the province increased from 809 in June to 10,119 in July, whereas those of *Cx. pipiens* decreased from 643 to 130 (Table).

**Table:** Entomological surveillance results, Milan Province, June–August 2026 (n = 11,817 specimens)

Species identified	Number of specimens		Total
	June	July	
*Aedes albopictus*	56	25	81
*Aedes caspius*	809	10,119	10,928
*Anopheles maculipennis*	9	0	9
*Anopheles maculipennis complex*	26	0	26
*Culex pipiens*	643	130	773
**Total**	**1,543**	**10,274**	**11,817**

Following the detection of human WNV clusters in Abbiategrasso and Peschiera Borromeo in July and early August, additional mosquito collections were performed using CDC-CO_2_ and BG-Sentinel traps. Pools of *Ae. albopictus, Ae. caspius, Cx. pipiens* and *Cx. modestus* tested negative for WNV and Usutu virus. Nevertheless, given the unusual abundance of *Ae. caspius*, archived RNA extracts from mosquitoes collected during routine surveillance in July in Milan and Pavia, the two provinces with most human cases, were retrospectively investigated. Seven *Ae. caspius* pools (ca 100 mosquitoes per pool) from three independent surveillance sites (two located in the Pavia Province and one in the Milan Province) were available for analysis. In six of the seven pools, WNV lineage 2 RNA was detected, with positive pools identified at all sites.

Mosquito identification and molecular analyses were performed using standard procedures employed within the Italian WNV surveillance network [6,7]. Confirmatory testing of the archived extracts was conducted at the National Reference Centre for Exotic Animal Diseases (CESME), thereby confirming the initial findings.

## Molecular characterisation and sequencing

To assess the possible role of *Ae. caspius* in increased WNV circulation, mosquito and human samples with sufficient viral load were whole genome sequenced after enrichment on the MiSeq platform (Illumina, Inc., United States). A total of 17 WNV sequences were obtained, comprising 16 human samples and one *Ae. caspius* mosquito pool, with a mean genome coverage of 95.7% (range: 74.4–100.0%) and mean depth ≥ 300 × (range: 50–1,000 ×). All sequences belonged to WNV lineage 2 (Supplementary Table 1).

For phylogenetic analyses, all the 17 sequences were aligned with 39 representative WNV lineage 2 genomes retrieved from the National Center for Biotechnology Information (NCBI) GenBank database. Maximum-likelihood phylogenetic reconstruction was performed using IQ-TREE v3.1.3 under the TIM3 + F + G4 substitution model selected by ModelFinder according to the Akaike information criterion (Figure 2). Node support was assessed using 1,000 ultrafast bootstrap replicates. The final alignment comprised 56 sequences and 10,125 nt positions. The *Ae. caspius*-derived genome clustered with contemporary human-derived genomes from Lombardy and shared > 99.94% nt identity with the three most closely related human sequences, differing by a maximum of three synonymous substitutions (Figure 2).

**Figure 2:**
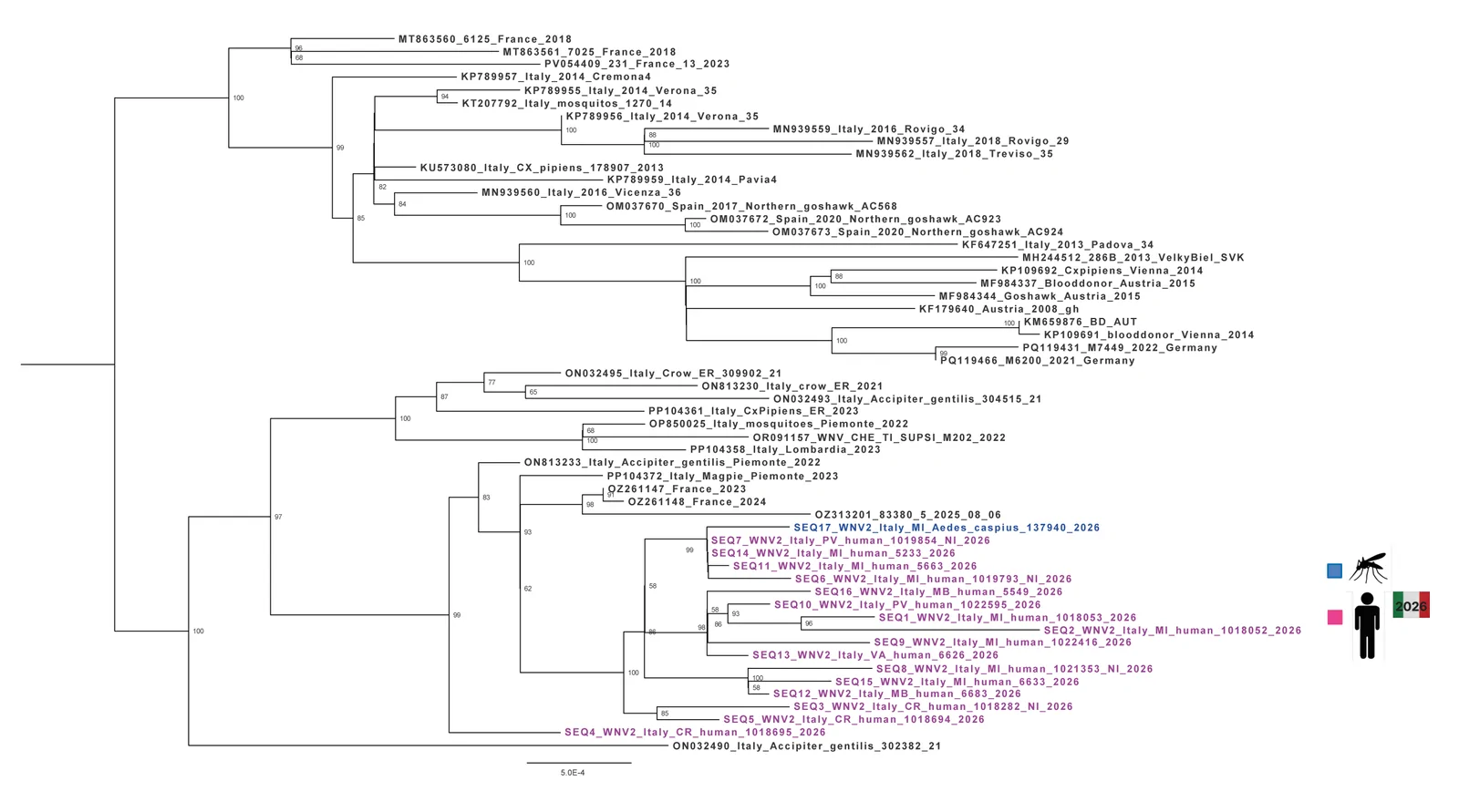
Maximum-likelihood phylogenetic tree showing the relatedness of selected WNV lineage 2 genome sequences, including those of WNV strains recovered in humans and *Aedes caspius* mosquitoes in Lombardy, Italy, 2026 (n = 56 strain sequences)

WNV: West Nile virus.

Sequences obtained from human cases in northern Italy in 2026 are highlighted in pink, while the sequence obtained from an *Ae. caspius* pool is highlighted in blue. Bootstrap support values > 50% are shown at the nodes. Sequence identifiers (Seq + number) correspond to those reported in Figure 1B and the Supplementary Table 1.

## Discussion

The 2026 WNV season in Lombardy was characterised by an unusually early onset of human cases of neuroinvasive disease, accompanied by evidence of viral circulation in birds, mosquitoes and horses. In northern Italy, where WNV has circulated endemically since 2008, *Cx. pipiens* is considered the principal vector, and transmission is monitored through integrated human, veterinary and entomological surveillance [1-5,8-10]. During the 2026 WNV transmission season, a marked shift in mosquito community composition was observed at several surveillance sites, with a > 12-fold increase in *Ae. caspius* abundance and a concurrent reduction in *Cx. pipiens*.

Mosquitoes of the *Ae. caspius* are widely distributed in Italy and may reach high densities in wetland and irrigated areas [11]. Although WNV RNA has previously been detected in field-collected *Ae. caspius* in Italy and elsewhere in Europe [9,10], evidence supporting its vector competence remains limited, particularly for lineage 2 viruses [12,13]. In this context, detection of WNV lineage 2 RNA in six of seven retrospectively analysed *Ae. caspius* pools from three independent sites is noteworthy. However, because these samples were selected retrospectively, positivity rates cannot be interpreted as estimates of infection prevalence.

Detection of WNV RNA alone does not demonstrate vector competence, as viral RNA may originate from infected blood meals without subsequent replication or dissemination. Therefore, our results do not establish a direct role for *Ae. caspius* in human WNV transmission.

The *Ae. caspius* derived genome clustered with contemporaneous human-derived genomes and shared > 99.94% nt identity, indicating circulation of closely related lineage 2 viruses during the same transmission season. While, genomic similarity cannot determine transmission pathways, repeated detection of WNV in *Ae. caspius* during an outbreak associated with early human cases warrants further investigation.

Several limitations should be considered when interpreting the current observations. First, the analysis of *Ae. caspius* pools was conducted retrospectively on archived material selected because of the unusual epidemiological situation, precluding estimation of infection prevalence or comparison with other mosquito species. Second, only one *Ae. caspius* derived genome was available for sequencing, limiting assessment of viral diversity within mosquito populations. Third, no virus isolation, tissue-specific analyses or experimental infection studies were performed; therefore, productive infection, viral dissemination and transmission competence could not be evaluated. Finally, host-feeding data and environmental determinants of mosquito abundance were unavailable.

Overall, findings should be interpreted as a temporal association between early human WNV transmission, enzootic virus circulation, unusually high *Ae. caspius* abundance and repeated detection of WNV RNA in this species. While *Cx. pipiens* remains the principal vector implicated in WNV transmission in northern Italy [5,8], the results highlight the value of investigating non-*Culex* mosquito species during atypical WNV transmission seasons. Further studies integrating mosquito infection rates, host-feeding patterns and environmental data may help clarify whether the observations described in this report reflect incidental infection or a reproducible epidemiological phenomenon.

## Conclusions

An unusually early WNV transmission season occurred in Lombardy in 2026, at a time when *Ae. caspius* mosquitoes were exceptionally abundant at several surveillance sites. In six of seven archived *Ae. caspius* pools collected during the season from three independent locations, WNV lineage 2 RNA was detected. The virus sequence obtained from one mosquito pool moreover clustered closely with human-derived sequences in phylogenetic analysis. While repeated WNV RNA detection in *Ae. caspius* during an early WNV season neither demonstrates productive infection, vector competence nor a role of this species in human transmission, this merits further investigations. Continued integrated surveillance, including targeted investigation of mosquito species other than *Cx. pipiens* during atypical epidemiological events, may help clarify the relevance of this finding.

## Data Availability

Sequences are submitted to the NCBI and GISAID public datasets with the following accession numbers. WNV2_Italy_MI_human_1018053_2026:QB136093; WNV2_Italy_MI_human_1018052_2026: QB136094; WNV2_Italy_CR_human_1018282_2026:QB136095; WNV2_Italy_CR_human_1018695_2026: QB136096; WNV2_Italy_CR_human_1018694_2026: QB136097; WNV2_Italy_MI_human_1019793_2026: QB136098; WNV2_Italy_PV_human_1019854_2026: QB136099; WNV2_Italy_MI_human_1021353_2026: QB136101; WNV2_Italy_MI_human_1022416_2026: QB136102; WNV2_Italy_PV_human_1022595_2026: QB136103; WNV2_Italy_MI_human_5663_2026: QB153277; WNV2_Italy_MB_human_6683_2026: QB153278; WNV2_Italy_VA_human_6626_2026: QB153279; WNV2_Italy_MI_human_5233_2026: QB153280; WNV2_Italy_MI_human_6633_2026: QB153281; WNV2_Italy_MB_human_5549_2026: QB153282; WNV2_Italy_MI_Aedes caspius_137940_2026: QB136208.

## Supplementary Data

335000 Supplementary File
