# Supplementary material for: Repeated detection of West Nile virus in Aedes caspius during an unusually early transmission season in humans in northern Italy, 2026
Source: Euro Surveill. 2026 Oct 22;31(42):2600862. doi: 10.2807/1560-7917.ES.2026.31.42.2600862 (PMC13695009; doi:10.2807/1560-7917.ES.2026.31.42.2600862)
Supplement: Supplementary file 1 [file 26-00862_MORENO_SupplementaryFile.pdf]

This supplementary material is hosted by *Eurosurveillance* as supporting information alongside the article **Repeated detection of West Nile virus in *Aedes caspius* during an unusually early transmission season in humans in northern Italy, 2026,** on behalf of the authors, who remain responsible for the accuracy and appropriateness of the content. The same standards for ethics, copyright, attributions and permissions as for the article apply. Supplements are not edited by Eurosurveillance and the journal is not responsible for the maintenance of any links or email addresses provided therein.

Supplementary Files

Supplementary Table 1- Characteristics of human and mosquito samples included in the sequencing. Sequence number correspond to those reported in Figure 1B and 2.

Sample	Sequenze no.	Submission date (DD-MM)	Municipality	Province	Material	Neuroinvasive form	% of coverage		% of identity to Aedes caspius seq.		GenBank Accession no
WNV2_Italy_MI_human_1018053_2026	1	16-07	Robecchetto Con Induno	Milano	Plasma	No	95.4	%	99.61	%	QB136093
WNV2_Italy_MI_human_1018052_2026	2	16-07	Robecchetto Con Induno	Milano	Urine	No	74.4	%	98.24	%	QB136094
WNV2_Italy_CR_human_1018282_2026	3	18-07	Malagnino	Cremona	Urine	Yes	99.8	%	99.81	%	QB136095
WNV2_Italy_CR_human_1018695_2026	4	21-07	Gadesco-Pieve Delmona	Cremona	Plasma	No	91.2	%	99.08	%	QB136096
WNV2_Italy_CR_human_1018694_2026	5	21-07	Gadesco-Pieve Delmona	Cremona	Urine	No	99.7	%	99.63	%	QB136097
WNV2_Italy_MI_human_1019793_2026	6	29-07	Abbiategrasso	Milano	Urine	Yes	98.6	%	99.91	%	QB136098
WNV2_Italy_PV_human_1019854_2026	7	30-07	Pavia	Pavia	Urine	Yes	99.9	%	99.95	%	QB136099
WNV2_Italy_MI_human_1021353_2026	8	10-08	Solaro	Milano	Urine	Yes	99.9	%	99.80	%	QB136101
WNV2_Italy_MI_human_1022416_2026	9	18-08	Peschiera Borromeo	Milano	Urine	Yes	88.6	%	99.81	%	QB136102
WNV2_Italy_PV_human_1022595_2026	10	19-08	Voghera	Pavia	Urine	No	94.3	%	99.69	%	QB136103
WNV2_Italy_MI_human_5663_2026	11	15-08	Rho	Milano	CSF*	Yes	99.0	%	99.95	%	QB153277
WNV2_Italy_MB_human_6683_2026	12	25-08	Muggio’	Monza Brianza	Urine	Yes	99.8	%	99.85	%	QB153278
WNV2_Italy_VA_human_6626_2026	13	25-08	Caronno Pertusella	Varese	Urine	No	100.0	%	99.86	%	QB153279
WNV2_Italy_MI_human_5233_2026	14	10-08	Cusago	Milano	Urine	No	99.9	%	99.94	%	QB153280
WNV2_Italy_MI_human_6633_2026	15	25-08	Solaro	Milano	Urine	Yes	99,8%	%	99.83	%	QB153281
WNV2_Italy_MB_human_5549_2026	16	13-08	Barlassina	Monza Brianza	Urine	No	100.0	%	99.84	%	QB153282
WNV2_Italy_MI_Aedes caspius_137940_2026	17	13-07	Rosate	Milano	Mosquitoes	\\	100.0	%	\\	%	QB136208

* CSF: Cerebrospinal fluid
